# The Role of Intravenous Anesthetics for Neuro: Protection or Toxicity?

**DOI:** 10.1007/s12264-024-01265-4

**Published:** 2024-08-17

**Authors:** Kaixin Wang, Yafeng Wang, Tianhao Zhang, Bingcheng Chang, Daan Fu, Xiangdong Chen

**Affiliations:** 1 Department of Anesthesiology, Union Hospital, Tongji Medical College, Huazhong University of Science and Technology, Wuhan, 430022 China; 2 Institute of Anesthesia and Critical Care Medicine, Union Hospital, Tongji Medical College, Huazhong University of Science and Technology, Wuhan, 430022 China; 3 Key Laboratory of Anesthesiology and Resuscitation, (Huazhong University of Science and Technology), Ministry of Education, Wuhan, 430022 China; 4 The Second Affiliated Hospital of Guizhou, University of Traditional Chinese Medicine, Guiyang, 550003 China

**Keywords:** Intravenous anesthetics, Neuronal injury, Neuroprotection, Neurotoxicity, CNS, PNS

## Abstract

The primary intravenous anesthetics employed in clinical practice encompass dexmedetomidine (Dex), propofol, ketamine, etomidate, midazolam, and remimazolam. Apart from their established sedative, analgesic, and anxiolytic properties, an increasing body of research has uncovered neuroprotective effects of intravenous anesthetics in various animal and cellular models, as well as in clinical studies. However, there also exists conflicting evidence pointing to the potential neurotoxic effects of these intravenous anesthetics. The role of intravenous anesthetics for neuro on both sides of protection or toxicity has been rarely summarized. Considering the mentioned above, this work aims to offer a comprehensive understanding of the underlying mechanisms involved both in the central nerve system (CNS) and the peripheral nerve system (PNS) and provide valuable insights into the potential safety and risk associated with the clinical use of intravenous anesthetics.

## Introduction

Intravenous anesthetics such as Dex, propofol, and ketamine have demonstrated neuroprotective effects in various neurological injury models. These mechanisms include the inhibition of inflammatory responses, apoptosis, oxidative stress, calcium overload, regulation of neurotransmitters, modulation of autophagy, and stabilization of cellular and synaptic structures [1–6]. A number of clinical studies have also confirmed the neuroprotective effects of intravenous anesthetics. For example, Dex has been shown to exert neuroprotective effects in epilepsy surgery [7], craniotomy [8], and carotid endarterectomy [9].

However, there is increasing evidence suggesting that general anesthetics may potentially cause long-term damage to the central nervous system, affecting neurogenesis and resulting in cognitive deficits [10, 11]. Some studies have investigated the effects of ketamine and propofol on neurotoxicity in developing rats [12, 13], which has been associated with cognitive dysfunction. The underlying mechanisms mainly include apoptosis, oxidative stress, calcium overload, inhibition of mitochondrial function, and disruption of fatty acid metabolism.

There is little evidence of peripheral nerve injuries (PNI) from intravenous anesthetics. Intravenous anesthetics used as adjuvants for peripheral nerve blocks are usually administered in two ways: intravenously and by perineural injection The relevant evidence mainly focused on the possibility of intravenous anesthetics as a local injection adjuvant to diminish the damage of peripheral nerve blocks caused by local anesthetics [14–17] and the possibility of ketamine and Dex in treating neuropathic pain [18–26]. By contrast, rare evidence has linked intravenous administration of anesthetics with PNI. Therefore, the evidence about the relationship between intravenous anesthetics and PNI is far from sufficient.

In general, the effects of intravenous anesthetics on neurological damage are not fully understood, and previous studies have confirmed the dual effects of these agents in terms of neuroprotection and neurotoxicity in both the CNS and PNS. Whether intravenous anesthetics can be safely used for neuroprotective treatment remains unanswered. Therefore, we aim to provide a comprehensive review of commonly used intravenous anesthetics such as Dex, ketamine, propofol, midazolam, etomidate, and remimazolam, assessing their role in both neuroprotection and neurotoxicity. Finally, we will provide insights into their potential for neuroprotective therapies.

## Dex

Dex is a highly selective α_2_-adrenergic receptor (α_2_-AR) agonist known for its sedative, anxiolytic, and analgesic effects while causing minimal respiratory depression [27]. It has been widely used in surgical procedures and in preventing postoperative psychosis [28]. In addition to its potent sedative effects, a growing body of animal studies has highlighted the potential of Dex to improve neurological outcomes following various brain and spinal cord injuries. The exact mechanism behind the neuroprotective effects of Dex in traumatic brain injury (TBI) remains unclear. However, recent evidence suggests that these cerebroprotective effects may not only be attributed to its α-2 agonist properties [29] but also involve its binding to imidazoline II receptors [30]. Furthermore, an increasing number of clinical trials have reported that Dex can reduce the incidence of postoperative neurological issues, such as delirium [31] and stroke [32], which underscores the promising potential of Dex as a neuroprotective agent in clinical practice.

**Fig. 1:**
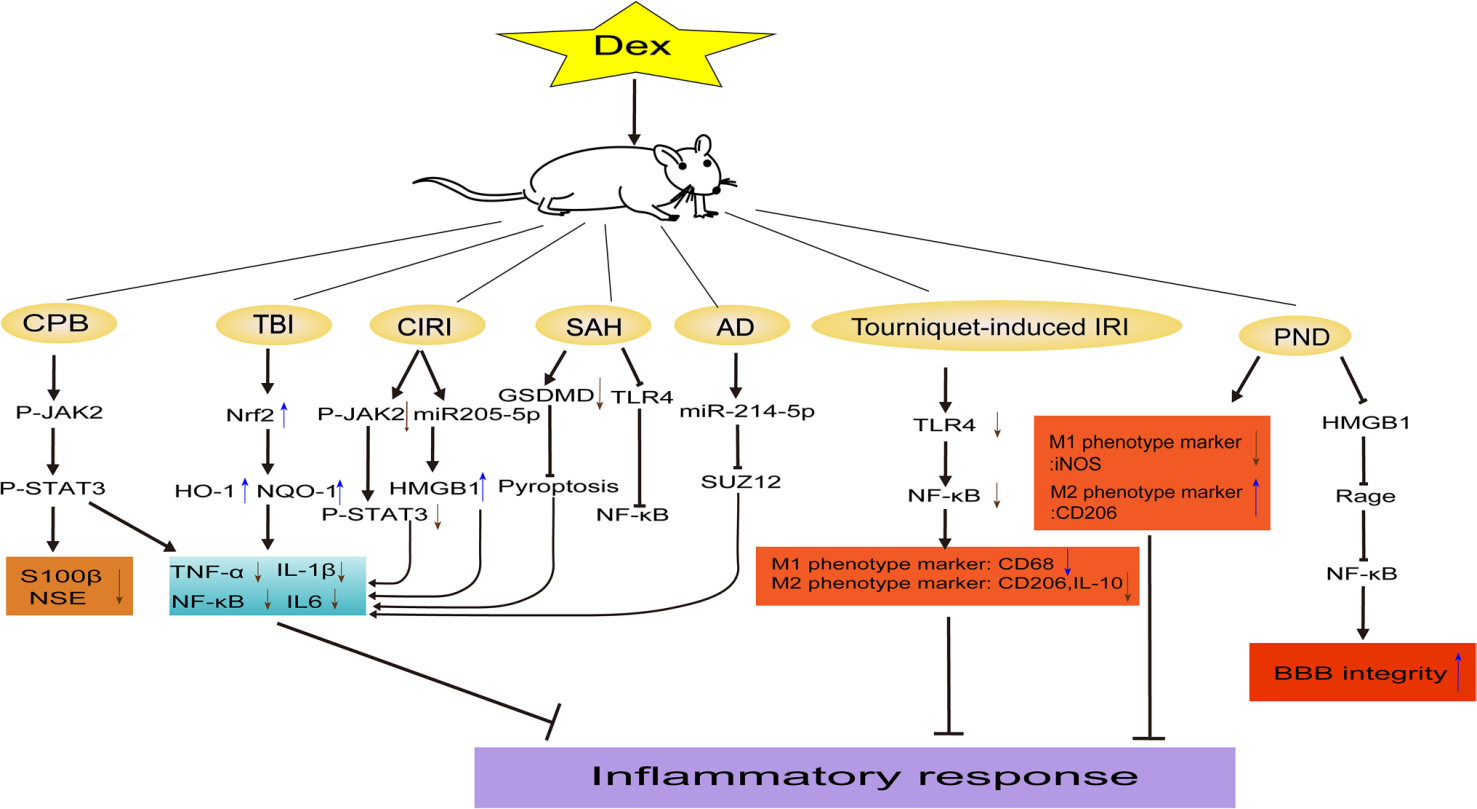
The inflammation-related neuroprotective mechanisms of Dex in different animal models. References: [49, 53, 59, 60, 215–219]. Abbreviations: Dex, Dex; CPB, cardiopulmonary bypass; TBI, traumatic brain injury; CIR, cord ischemic reperfusion; SAH, subarachnoid hemorrhage; AD, Alzheimer's disease; IRI, ischemic reperfusion injury; PND, postoperative neurological dysfunction; p-JAK2, phosphorylation-Janus kinase 2; p-STAT3, phosphorylation-signal transducer and activator of transcription 3; S100β, S100 calcium-binding protein β; NSE, neuron-specific enolase; Nrf2, Nuclear Factor erythroid 2-Related Factor 2; HO-1, Heme Oxygenase-1; NQO-1, NAD(P)H quinone oxidoreductase-1; TNF-α,tumor-necrosis factor-α; NF-κB, Nuclear Factor- Kappa B; IL-6, interleukin-6; HMGB1, high-mobility group box 1; GSDMD, Gasdermin domain; TLR-4, toll-like receptor-4; SUZ12, suppressor of zeste 12; iNOS, Inducible Nitric Oxide Synthase; Rage, receptor for advanced glycosylation end products; BBB, blood-brain barrier. **Note:**↓**,** promote;  , inhibit;↑,up-regulation;↓,down-regulation.

**Fig. 2:**
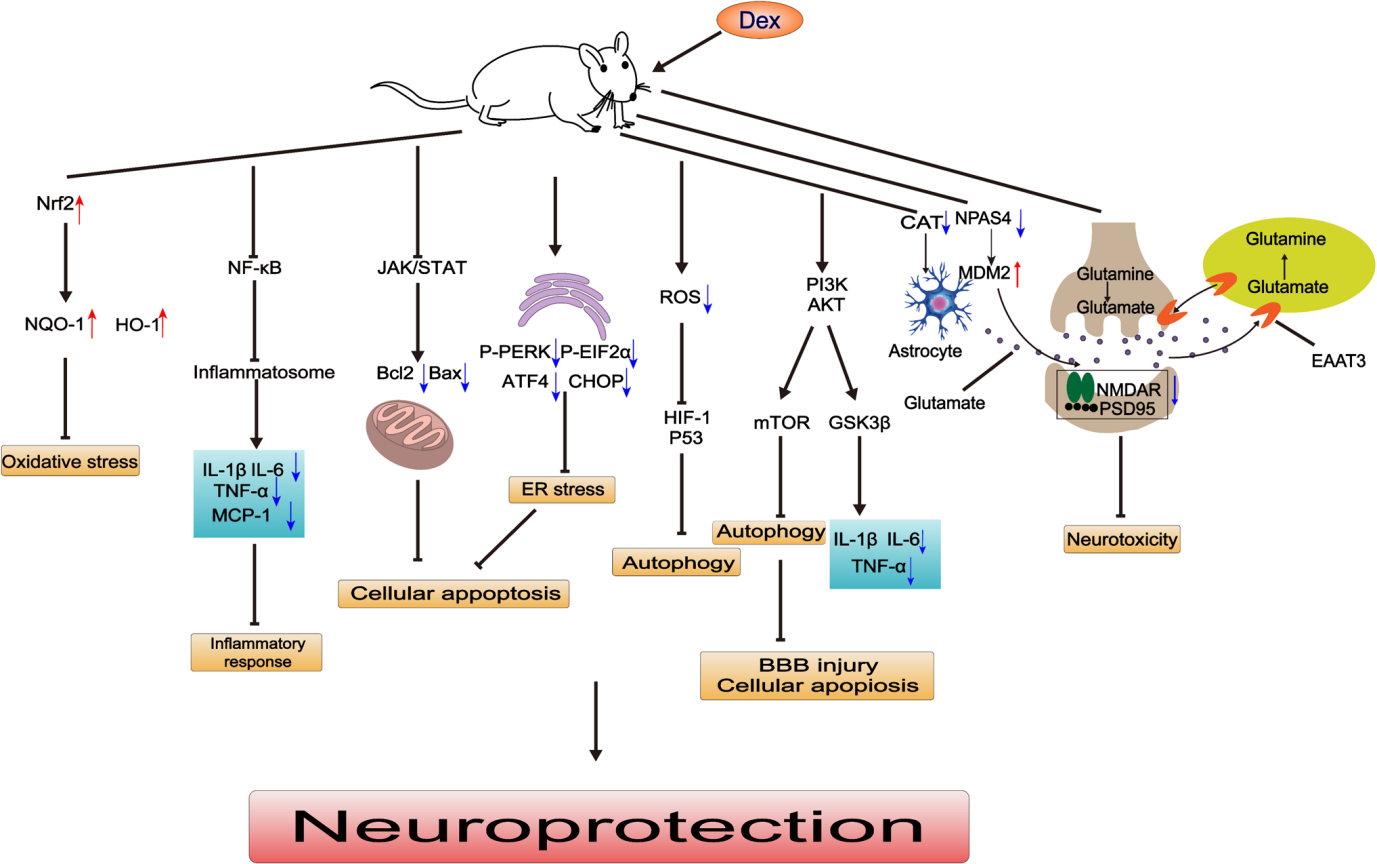
The neuroprotective mechanisms of Dex. References: [1, 29, 44, 56, 63, 65, 70, 71, 220]. Abbreviations: Dex, Dex; Nrf2, Nuclear Factor erythroid 2-Related Factor 2; NQO-1, NAD(P)H quinone oxidoreductase-1; HO-1, Heme Oxygenase-1; NF-κB, Nuclear Factor- Kappa B; IL-1β, interleukin-1β; TNF-α, tumor-necrosis factor-α; MCP-1, Monocyte chemoattractant protein-1; JAK, Janus kinase; STAT, signal transducer and activator of transcription; Bcl2, B cell lymphoma/leukemia 2; Bax, Bcl2 Associated X Protein; ER, endoplasmic reticulum; p-PERK, phosphorylation-protein kinase R (PKR)-like ER kinase ; P-EIF2α, phosphorylation-eukaryotic translation initiation factor 2 alpha; ATF4, activating transcription factor 5; CHOP, C/EBP homologous protein; ROS, reactive oxygen species; HIF-α, hypoxic-induced factor-α; P53, tumor suppressor gene; PI3K, Phosphatidylinositol3-kinase; AKT, Protein Kinase B(PKB); mTOR, Mechanistic Target Of Rapamycin; GSK3β, Glycogen Synthase Kinase 3 Beta ; BBB, blood brain barrier; CAT, catecholamine; NPS4, neuronal PAS domain protein 4; MDM2, mouse double minute 2 homolog; NMDAR, N-methyl-D-aspartate-receptor; PSD95, postsynaptic density protein-95; EAAT3, Excitatory Amino Acid Transporter 3. **Note**: ↓**,** promote;  , inhibit;↑,up-regulation;↓,down-regulation.

Dex could exhibit protective effects on various organs, including the heart (cardioprotection), kidneys (renoprotection), brain (cerebral protection), and lungs (pulmonary protection) [6, 33–35]. In terms of neuroprotection, an increasing body of research indicate that Dex mitigates neuronal injury and enhances neurological function in various models, including hypoxic neuronal injury model [36], ischemia-reperfusion injury model [37], cerebral hemorrhage model [38], post-traumatic brain injury [1], anesthetic neuronal injury [10, 39], substance-induced neuronal injury [6, 40], epilepsy and neurodegeneration [41, 42]. The neuroprotective effects of Dex are attributed to a wide range of mechanisms, including the regulation of neurotransmitters, modulation of inflammatory responses, oxidative stress, apoptotic pathways, autophagy, mitochondrial function, and engagement in other cellular signaling pathways. The summary of these neuroprotective mechanisms of Dex has been concluded (Figs. 1, 2).

### Regulation of Neurotransmitters

Neurotransmitters play a crucial role in various physiological processes, including signal transduction, the release of hormones and peptides, as well as cellular responses to damage. Excitatory neurotransmitters like glutamate [43] have been implicated in nerve damage. The regulation of glutamate release has been shown to play an important role in the neuroprotection of Dex. Dex regulates the release of glutamate by reducing the release of catecholamines (CAT) [44]. Furthermore, Dex could significantly increase the oxidative metabolism of glutamate in astrocytes and block the conversion of glutamate to glutamine, thus mitigating glutamate-induced neurotoxicity [45]. Excitatory amino acid transporter 3 (EAAT3), a member of the glutamate transporter family, plays a vital role in removing glutamate from the synaptic gap through the reuptake of glutamate by glial cells and neurons. It has been suggested that Dex could enhance the expression of EAAT3 [46], thereby protecting against neuronal injury caused by isoflurane.

In conclusion, the regulation of neurotransmitters, especially glutamate, may play a vital role in the neuroprotective effects of Dex.

### Inflammatory Response

The significance of the immune or inflammatory response is not fully understood. On one hand, it exacerbates oxidative stress, on the other hand, phagocytes kill or destroy nerve cells and help to maintain neurogenesis. Inflammation or neuroinflammation, is therefore a complex process. In recent years, numerous studies have shown that the inflammatory response is closely linked to the neuroprotective effects of Dex. Microglia and macrophages play an important role in acute neuroinflammation. Dex could inhibit the reactivity of microglia, thus reducing the release of inflammatory factors like interleukin-1β (IL-1β), Tumor necrosis factor-α (TNF-α), interleukin-6 (IL-6), and monocyte chemotactic protein 1 (MCP-1) [1]. Ding *et al.* showed that Dex treatment could reduce the expression of CD40 and CD86, suggesting an inhibitory effect on the activation and infiltration of macrophage [47, 48]. Dex pretreatment could also decrease the expression of the pro-inflammatory marker M1 cells and upregulate the expression of the M2 phenotypic markers arginase 1 and CD206 [49]**.** Many studies have indicated that Dex inhibited the formation of inflammatory vesicles and the expression of inflammatory factors such as Nod-like receptor thermal protein (NLRP3) and caspase-1, resulting in improved performance in the Morris water maze (MWM) test in rats [1]. Dex may also exert neuroprotective effects by inhibiting the activation of inflammatory vesicles and attenuating the toxic effects of local anesthetics [50]. Similarly, a meta-analysis of 879 patients has confirmed the neuroprotective effects of Dex in suppressing the inflammatory response, reducing neuroendocrine hormone release, and maintaining intracranial homeostasis [51].

In a word, many studies have shown that the inflammatory response is closely linked to the neuroprotective effects of Dex (Fig. 1).

### Oxidative Stress

Oxidative stress plays an important role in the inflammatory response. Nuclear factor erythroid 2-related factor 2 (Nrf2) is a vital transcription factor that regulates the antioxidant activity of cells, inhibits oxygen species (ROS) escalation, and reduces the formation of macrophages into the pro-inflammatory M1 phenotype [52]. Nicotinamide adenine dinucleotide phosphate-quinone oxidoreductase-1 (NQO-1) and heme oxygenase-1 (HO-1) are essential antioxidant enzymes that could alleviate cellular damage caused by oxidative stress by promoting the clearance of ROS. Research has demonstrated that Dex can increase the expression of Nrf2 and its downstream proteins, NQO-1 and HO-1, in the brain tissue of TBI rats, thereby exerting neuroprotective effects [53]. Dex can confer neuroprotection by activating Nrf2, which, in turn, could suppress the expression of NLRP3, leading to reduced levels of IL-1β and other inflammatory mediators [54]. Additionally, studies have indicated that in hypoxia-activated BV2 microglia and hypoxic neonatal rats, Dex could reduce synaptic loss and neuronal damage in the hippocampus, and the underlying mechanism may be attributed to its ability to prevent hypoxia-induced activation of nicotinamide adenine dinucleotide phosphate oxidases (NOX2) in microglia, thereby inhibiting oxidative stress and neuroinflammatory responses [55].

### Apoptosis and Pyroptosis

The anti-apoptotic protein B-cell lymphoma-2 (Bcl2) and pro-apoptotic protein Bcl-2 associated X (Bax) play crucial roles in the mitochondrial apoptosis pathway. Dex was found to inhibit apoptosis by upregulating the expression of the anti-apoptotic protein Bcl2 and heat shock protein 70 (HSP70) while decreasing the expression of Bax [2]. Endoplasmic reticulum stress and oxidative stress contribute to apoptosis. It was observed that Dex could reduce the levels of endoplasmic reticulum stress markers, including phospho-PERK (p-PERK), phosphor-EIF2α (p-EIF2α), activating transcription factor 4 (ATF4), and C/EBP-homologous protein (CHOP) after TBI [56]. Several pathways, such as the PERK-CHOP-Caspase-11 pathway, PERK-ATF4-CHOP signaling pathway [56], CNPY2-PERK(CNPY2, canopy homolog 2; PERK, PKR-like ER kinase) [57] and Sig-1R/GRP78/CHOP/Caspase-3 pathway(Sig-1R, sigma-1 receptor; GRP78, glucose-regulated protein-78) [58], in the endoplasmic reticulum, are involved in the neuroprotective effects of Dex. In the oxygen-glucose deprivation (OGD) model, Dex can regulate the expression of apoptosis-related proteins by suppressing the JAK/STAT (JAK, Janus Kinase; AKT/PKB, protein kinase B) pathway [59]. Additionally, Dex has demonstrated its ability to inhibit apoptosis induced by oxidative stress while promoting the repair of synaptic damage by inhibiting the interaction between N-Methyl-D-aspartic acid (NMDA) and postsynaptic density protein 95 (PSD95), thus reducing the formation of NMDA-PSD95 complexes [58].

Apart from its impacts on cellular apoptosis, Dex also played a role in cellular proptosis in neuroprotection, which has been shown to reduce early brain injury in rats with subarachnoid hemorrhage by inhibiting the activation of TLR4/NF-κB(TLR4, toll-like receptor 4; NF-κB, nuclear factor-kappa B) pathway and attenuating eosinophilic prolapse of microglia [60]. Moreover, in a sepsis model, Dex could exert neuroprotective effects by inhibiting the proptosis of astrocytes [6].

In summary, apoptosis and pyroptosis may play an important role in the neuroprotective effects of Dex.

### Autophagy

Autophagy is a cellular mechanism that helps to maintain normal tissue homeostasis by degrading and recycling aged, excess, or dysfunctional proteins [61]. Autophagy (a double-edged sword [62]) has been reported to have a dual role in the neuroprotection of intravenous anesthetics. Dex is reported to affect autophagy-related signaling pathways. Dex could reduce the expression of autophagy-related proteins Beclin-1 and microtubule-associated protein 1 light chain 3I/II(LC3I/II) in TBI in rats, attenuating the disruption of the blood-brain barrier, brain edema, and apoptosis [63]. Dex could reverse the up-regulated expression of low-density lipoprotein receptor-related protein 1b(Lrp1b) and DNA damage-regulated autophagy modulator 2(Dram2) while down-regulating the expression of miR-27A-3p, thereby inhibiting excessive autophagy and ameliorating the neurological damage caused by brain injury [64]. Dex also exerts neuroprotective effects through the activation of autophagy via the adenosine 5‘-monophosphate (AMP)-activated protein kinase (AMPK)-adrenergic receptor α pathway [27]. In addition, activation of PI3K/Akt/mTOR (PI3K, phosphatidylinositol 3-kinase; mTOR, mechanistic target of rapamycin), Nrf2/NQO-1/HO-1, HIF-1α (HIF, hypoxia inducible factor 1α) pathways [65–67] is closely related to autophagy.

### Nerve Regeneration and Cell Structural Protection

Neural regeneration also plays an important role in neuroprotection of Dex. It has been discovered that Dex can safeguard neurons and astrocytes against Aβ cytotoxic damage by stimulating the production of brain-derived neurotrophic factor (BDNF) [68]. Furthermore, it has been proposed that Dex could facilitate neuroregenerative recovery by enhancing the expression of BDNF secreted in a time and dose-dependent manner. Notably, the α2 adrenergic receptor antagonist yohimbine, but not the α1 receptor blocker prazosin, could thwart the neuroprotective effects of Dex. This suggests that the neuroprotective actions of Dex are mediated through an α2 receptor-dependent pathway [69]. In the context of neurons treated with OGD, it is suggested that the neurodegenerative regulator histone Deacetylase 5 (HDAC5) may promote the expression of murine double minute 2(MDM2) (an E3 ubiquitination ligase) by inhibiting the NPAS4 (neuronal PAS domain protein 4). Dex, in this context, exerts neuroprotective effects against cerebral ischemic injury by interfering with mdm2-induced ubiquitination and degradation of PSD-95 through the modulation of HDAC5 and NPAS4 [70]. Additionally, Zhao *et al*. demonstrated that the expression of PSD95 significantly decreased following TBI. Dex could reduce the formation of PSD95-NMDA complexes and promote the recovery of damaged synapses and cognitive impairment [71]. Additionally, Dex has been reported to protect the integrity of the membrane and nuclear structures in astrocytes and attenuate TBI-induced axonal damage [6].

Whatever, Dex could protect astrocytes and neurons by promoting nerve regeneration and cell structural protection.

## Propofol

Propofol, an intravenous anesthetic known for its rapid onset of action and short awakening time [72], exerts its effects by promoting the activation of the inhibitory neurotransmitter gamma-aminobutyric acid (GABA) receptor [73]. It’s widely used in neurosurgery anesthesia due to its ability to reduce cerebral blood flow, decrease cerebral oxygen consumption, and decrease intracranial pressure, which could exert powerful convulsive effects [74]. Studies have reported that propofol has both neuroprotective and neurotoxic effects.

### Neuroprotection

**Fig. 3:**
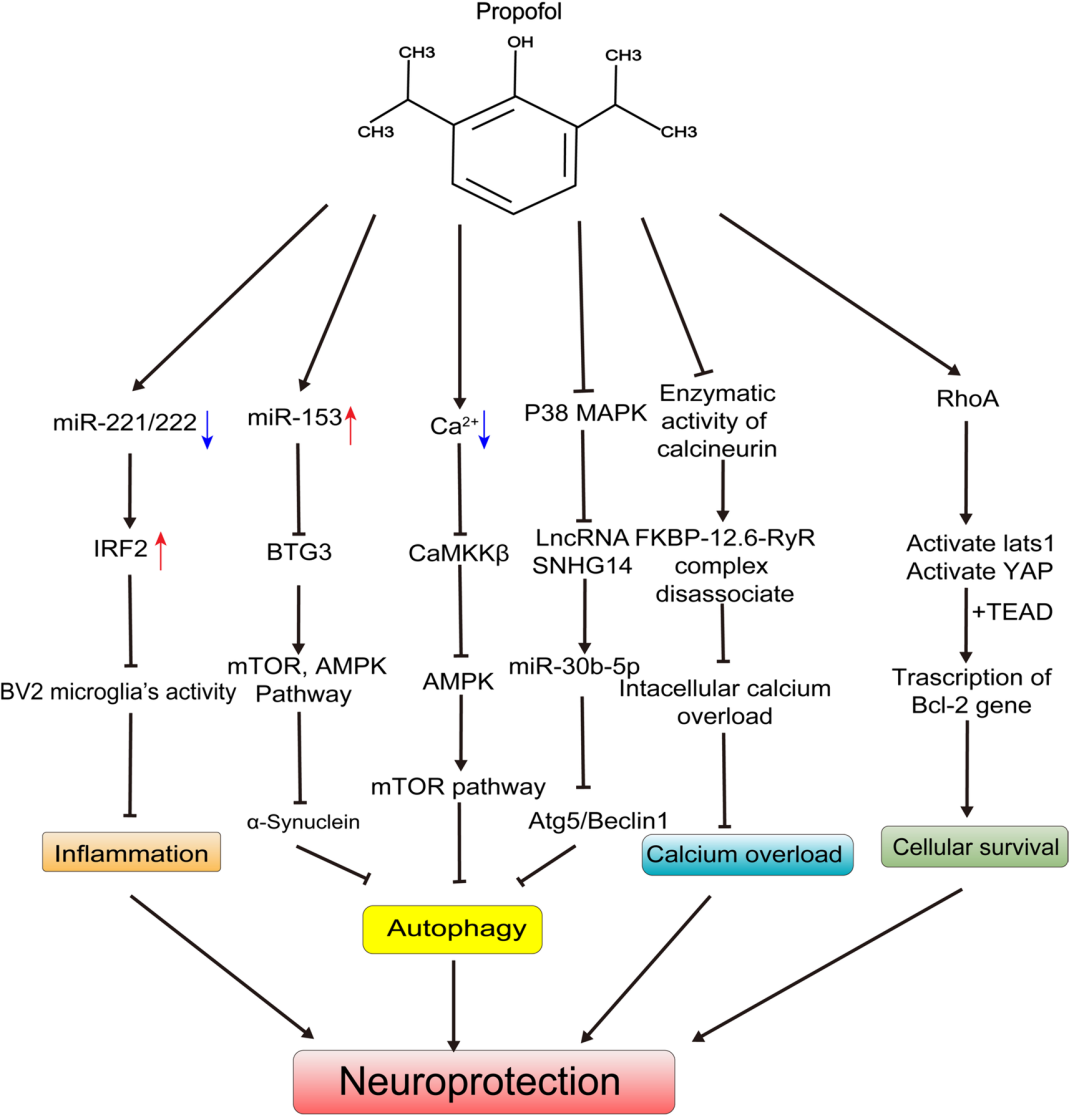
The neuroprotective mechanisms of propofol. References: [76, 89, 90, 92, 93]. Abbreviations: IRF2, interferon regulatory factor 2; BTG3, BTG family member 3; mTOR, Mechanistic Target of Rapamycin; AMPK, Adenosine 5‘-monophosphate (AMP)-activated protein kinase; HIF-α, hypoxic-induced factor-α; CaMKKβ, calmodulin-dependent protein kinase β; p38 MAPK, p38 MAP kinase; lncRNA, long non-coding RNA; ATG5, Autophagy Related Protein 5; FKBP-12.6, FK506 binding protein 12.6; RYR, ryanodine receptor; RhoA, Ras homolog gene family, member A; lats1, large tumor suppressor kinase 1; YAP, Yes-associated protein; TEAD, TEA domain family member; BCL2, B cell lymphoma/leukemia 2. **Note**: ↓**,** promote;  , inhibit;↑, up-regulation;↓, down-regulation.

Propofol possesses potent antioxidant properties. An increasing body of studies has shown that propofol shows neuroprotective effects in different models, including the ischemia-reperfusion model, traumatic brain injury, and hypoxic cellular models. The main neuroprotective mechanisms of propofol are related to autophagy, oxidative stress, inflammatory response, calcium overload, apoptosis, and mitochondrial damage. The neuroprotective mechanisms of propofol are concluded (Fig. 3).

#### *Inflammatory Response*

Inflammatory responses are pivotal in the neuroprotective action of propofol. To illustrate, propofol exerts its anti-inflammatory effects by hindering the production of pro-inflammatory mediators, including NO, TNF-α, and IL-6. It accomplishes this by down-regulating the expression level of miR155, which targets the suppressor of the cytokine signaling 1 (SOCS1) signaling pathway [75]. In the sepsis lipopolysaccharide (LPS) model, propofol could suppress the production of pro-inflammatory mediators by down-regulating the expression levels of miR-221/222 [76]. Furthermore, propofol’s neuroprotective role in the stroke model involves the inhibition of the NLRP1-caspase-1-caspase-6 inflammatory pathway [77]. Additionally, propofol demonstrates an inhibitory effect on the expression of transglutaminase 2 (TGM2) in BV2 cells and microglia, leading to the suppression of NF-κB signaling and microglia activation [78]. Beyond these mechanisms, the inhibition of various other inflammatory signaling pathways, such as NF-κB, HIF-α, p38 mitogen-activated protein kinase (MAPK), and JAK1/STAT3 pathways [79–81], also play important roles in neuroprotection.

#### *Apoptosis*

Animal models have shown that propofol significantly protects cells from apoptosis induced by OGD. The mechanism may be the increasing expression of Bcl2 and the decreasing expression of cleaved caspase-3, phosphorylated SAPK/JNK (SAPK, stress-activated protein kinase; JNK, Jun N-terminal kinase), and phosphorylated c-Jun [82]. Furthermore, a study has indicated that propofol could attenuate the decreased cell survival rates and the increased apoptosis states induced by hypoxia. This effect is associated with the miR-153/BTG3/mTOR pathway (BTG3, B-cell translocation gene 3) [83]. In a preliminary double-blind, prospective, randomized study involving 44 patients with severe aneurysmal subarachnoid hemorrhage (ASAH), it was observed that intraoperative cerebrospinal fluid caspase-3 levels were reduced [84]. This suggested that general anesthesia may play a role in inhibiting the apoptotic pathway in neuronal cells.

#### *Autophagy*

Autophagy plays an important role in the neuroprotection/neurotoxicity of propofol. Autophagy represents a double-edged sword, as it has been demonstrated that propofol can exert neuroprotective effects by either inhibiting hippocampal autophagy or promoting it [4, 85]. Furthermore, a close connection exists between autophagy and apoptosis. It has been observed that the activation of autophagy can trigger an apoptotic cascade, ultimately leading to neuronal cell damage [86]. Conversely, it has also been established that excessive autophagy can safeguard neurons against apoptosis in neurodegenerative diseases, playing a protective role against neuronal cell damage [87]. Consequently, autophagy is a process that requires meticulous regulation. Research has indicated that overexpression of neuronal PAS domain protein 4 (NAPS4) could reduce propofol-induced neurotoxicity by inhibiting autophagy in hippocampal neuronal HT22 cells [88]. Another study has affirmed that propofol counteracts neuronal damage induced by OGD, possibly mediated through the inhibition of autophagy via the Ca^2+^/CaMKKβ/AMPK/mTOR pathway (CaMKKβ, calmodulin-dependent protein kinase β) [89]. Additionally, propofol could inhibit the expression of long non-coding RNA (lncRNA) SNHG14 through the p38 MAPK signaling pathway, thereby suppressing autophagy and mitigating cerebral ischemia-reperfusion injury (CIRI) [90]. Although autophagy is a double-edged sword, many studies have shown the significant role of autophagy in the neuroprotective effects of propofol.

#### *Oxidative Stress*

Propofol was also found to mitigate neuronal oxidative damage. Its use in the oxidative damage model group was associated with the increased expression of mitochondrial fusion proteins and anti-apoptotic proteins, and the decreased expression of apoptotic proteins [91]. Moreover, propofol proficiently attenuated oxidative stress injuries, resulting in a substantial elevation in the activity of Superoxide Dismutase (SOD) and Glutathione Peroxidase (GPX) in rat brain tissue, while concurrently reducing the levels of Malondialdehyde (MDA) and protein carbonyl compounds.

#### *MicroRNAs*

MicroRNAs also play an essential role in the neuroprotection of propofol. A study has indicated that propofol can increase Interferon Regulatory Factor 2 (IRF2) protein levels, thereby producing neuroprotective effects through the MiR-221/222/ IRF2 pathway [76]. MiR-153 can also down-regulate the expression of BTG3, and alleviate the inhibition of the mTOR pathway and the activation of the AMPK pathway, thus protecting PC-12 cells from being damaged by hypoxia [83]. The activation of the mTOR and AMPK pathways is closely related to neuroprotection. Propofol can inhibit autophagy following stroke by activating the mTOR/ Ribosomal protein S6 kinase β1 (S6k1) signaling pathway and reducing α-synaptic nuclear protein levels [92].

#### *Calcium Overload*

Calcium overload also plays a role in propofol’s neuroprotection. Propofol could safeguard hippocampal neurons from ischemic/reperfusion (I/R) injury via two independent signaling pathways: the calcium-regulated neurophosphatase/ calstabin2 (FKBP12.6) -RyR/calcium overload pathway and the RhoA/Lats1/Yap/Bcl2 pathway [93] (RhoA, Ras homolog gene family member A; Lats1, large tumor suppressor homolog 1; Yap, yes-associated protein). Additionally, it has been demonstrated that propofol's protective effect against hypoxia-induced inflammation and apoptosis in BV2 microglia is associated with intracellular Ca^2+^ homeostasis and the JAK1/STAT3 pathway [4].

#### *Astrocytes and Microglia*

Exposure to propofol following injury could lead to apoptosis of neuronal cells without causing apoptosis of astrocytes or microglia. There is substantial evidence confirming that propofol's neuroprotective effects are linked to astrocytes. For instance, propofol shields astrocytes from t-butyl hydroperoxide (t-BOOH)-induced intracellular acidification by maintaining the activity of Na^+^/H^+^ exchange pump [94]. In the hippocampal brain tissue sections of hypoxic rats, propofol could reduce the concentration of excitatory amino acid (EEA) in a dose-dependent manner, thereby inhibiting neuronal apoptosis and edema [95]. This mechanism may be related to its effects on the expression and function of astroglia water channel protein [96]. Moreover, propofol could protect regulatory T Cells, suppress neurotoxic astrogliosis, and potentiate neurological recovery after ischemic stroke [97]. Additionally, the activation of microglia also plays a role in propofol's neuroprotection, potentially by downregulating the expression of connexin 43 (Cx43) in microglia [98]. In conclusion, much evidence has confirmed that propofol's neuroprotective effects are linked to astrocytes and microglia.

### Neurotoxicity

Despite the strong neuroprotective effects demonstrated by propofol in basic and clinical trials, there are also studies showing its neurotoxic effects and cognitive impairment. Evidence of propofol's toxicity in humans comes from a phenomenon known as "propofol infusion syndrome" (PRIS). One of the independent risk factors for PRIS is traumatic brain injury (TBI) [99]. As a rare side effect of propofol, PRIS is usually observed in patients who use high doses (higher than 4mg/kg/h) of propofol for a prolonged period of time (>48 hours) [99]. A study revealed that propofol promoted mitochondrial damage in hippocampal neurons in a dose-dependent manner. Subanesthetic doses of propofol were neuroprotective in cerebral ischemia-reperfusion rats, but high doses of propofol had no effect. Additionally, some studies have confirmed the carcinogenic effects of propofol [100]. Additionally, neuronal apoptosis is considered one of the most widely studied neurotoxic outcomes resulting from exposure to propofol [101]. Studies have reported that propofol could trigger apoptosis via the cAMP/CREB (cAMP; cyclic adenosine monophosphate; CREB, cAMP response element binding protein) signaling pathway [102]. Hypoxic preconditioning (HPC) has been reported to attenuate propofol-induced neuronal apoptosis by increasing cAMP levels and CREB phosphorylation, thereby preventing caspase-3-induced apoptosis in hippocampal neurons [102]. It has also been suggested that propofol-induced hippocampal neuronal cell damage in developing rats and that erythropoietin could attenuate this damage by inhibiting the expression of TLR4, Bax, caspase-3, TLR4, and p65 [12]. Furthermore, propofol has also been reported to induce neuronal death in the developing brain, leading to cognitive dysfunction, including learning, memory, planning, and reasoning impairment [10]. It has been demonstrated that propofol induces brain dysfunction in young and adult rats by activating γ-GABA receptors and decreasing the expression of neurotransmitters and BDNF [5, 103, 104]. Furthermore, chronic exposure to propofol in aged rats can lead to long-term learning and memory impairment [105]. Learning and memory were impaired in pregnant rats through the BDNF-protomyosin receptor kinase B (TrkB) pathway, as well as through inhibiting the expression of Kinesin superfamily 17 (KIF17) and translocation of N-methyl-D-aspartate receptor subunit 2B (NR2B) to neuronal membranes [106]. It has also been suggested that propofol appears to exacerbate injury-induced apoptosis through the superinduction of the p75 neurotrophin receptor (p75NTR) and the increased pro-BDNF: mBDNF ratio, leading to a massive disruption of the homeostasis of pro-apoptotic/pro-survival neurotrophic factor signaling [107].

## Ketamine

**Fig. 4:**
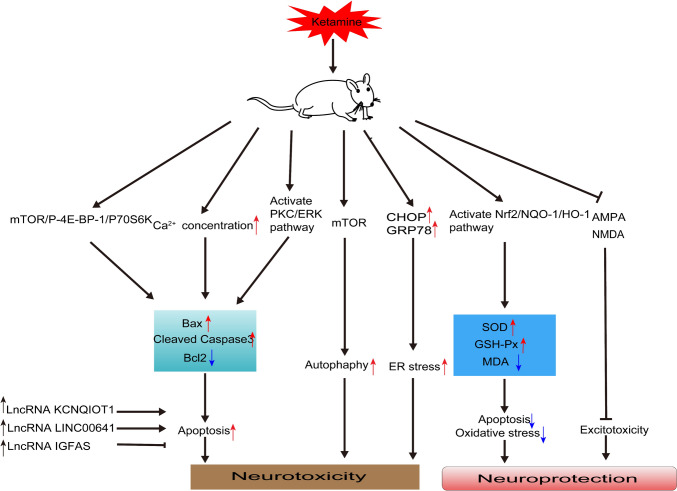
Neurotoxic and neuroprotective mechanisms of ketamine. References: [114, 122, 124, 221–224]. Abbreviations: ket, ketamine; mTOR, Mechanistic Target of Rapamycin; P-4E-BP-1, Phosphorylated 4E-binding protein 1; P70S6K, ribosomal protein 70 S6 kinase; lncRNA, long non-coding RNA; Bax, Bcl2 Associated X Protein; PKC, protein kinase C; BAX, Bcl-2 Associated X Protein; Bcl2, B cell lymphoma/leukemia 2; PKC, protein kinase C; ERK, extracellular regulated protein kinases; mTOR, Mechanistic Target of Rapamycin; CHOP, C/EBP homologous protein; GRP78, glucose-regulated protein 78; ER, endoplasmic reticulum; Nrf2, Nuclear Factor erythroid 2-Related Factor 2; HO-1, Heme Oxygenase-1; NQO-1, NAD(P)H quinone oxidoreductase-1; SOD, superoxide dismutase; GSH-PX, glutathione peroxidase; MDA, Malondialdehyde; AMPA, α-amino-3-hydroxy-5-methyl-4-isoxazole-propionicacid receptor; NMDA, N-methyl-D-aspartate. **Note**: ↓, promote;  , inhibit;↑,up-regulation;↓,down-regulation.

Ketamine is a non-competitive N-methyl-D-aspartate receptor blocker commonly used in pediatric anesthesia. However, ketamine began to be abused in the late 1970s and its side effects and potential for abuse have limited its wider and routine clinical use [108]. In recent years, researchers have focused on its rapid antidepressant effects [109], exploring its potential in treating various neuropsychiatric syndromes [110, 111]. In animal models, ketamine appears to exhibit both neurotoxic and neuroprotective properties (Table 1, Fig. 4). When administered at anesthetic doses during the neurodevelopmental stage, ketamine can stimulate inflammation, autophagy, apoptosis, and elevate reactive oxygen levels, resulting in neurotoxic effects. However, recent research indicates that the use of subanesthetic doses of ketamine may offer a potential new approach for treating acute neuronal injuries, neurodegenerative diseases, and other neuropsychiatric disorders [112, 113].

**Table 1:** The neuroprotective/neurotoxic effects of ketamine in different models of animals.

References	Models	Ketamine dose	Results
[11]	Postoperative neurological dysfunction (PND)	40 mg/kg	Ketamine exposure decreased cell proliferation in the subventricular zone (SVZ) and subgranular zone (SGZ), inhibited neural stem cell (NSC) proliferation and neuronal differentiation, promoted NSC apoptosis, and led to adult cognitive deficits
[201]	OGD	Ketamine preconditioning (10 μmol/L) in slices of hippocampal	Hypothermic preconditioning but not ketamine reduces OGD-induced neuronal injury correlated with downregulation of cyclooxygenase-2 (COX-2) expression in mouse hippocampal slices
[114]	—	0,50,100 and 500 μmol/L	Ketamine could dose-dependently promote the apoptosis of rat hippocampal neurons with upregulation of p-mTOR and its downstream regulators (p-4E-BP-1 and p-p70S6K)
[119]	Vitro model of neural stem cells-derived neurons (nSCNs)	50 μmol/L	Ketamine (50μM) caused markedly nSCNs apoptosis and neurite degeneration in vitro
[117]	Pregnant rats	40 mg/kg, a rate of 40–60 mg/kg per hour for 3 hours	Maternal anesthesia with ketamine in the second trimester of pregnancy can lead to cognitive memory impairment and neurotoxicity in the hippocampus of offspring through the Wnt/ β-catenin signaling pathway
[120]	Recombinant AAV (rAAV) injection into the hippocampus	7.5,15,30 mg/kg	Ketamine induced the apoptosis of neurons of the hippocampus in rats, and the apoptosis of PC-12 cells, accompanied by the down-regulation of KCNQ1OT1 and BDNF expressions, and up-regulation of miR-206 expression
[202]	Retinal ischemia/reperfusion model	0.118 mmol/L	Ketamine is safe for intravitreal use in doses up to 0.118 mmol·L^−1^ which seems to be particularly efficient in protecting the retina from ischemic injury
[125]	Status epilepticus (SE) model	40 mg/kg, subcutaneously	Ketamine and dantrolene inhibited electroconvulsive seizures (ECS)-induced apoptosis and non-apoptotic injury, they did not produce similar effects on memory retention
[129]	Traumatic brain injury (TBI) model	30 mg/kg per day	Ketamine dramatically increased microglial proliferation in the granule cell layer of the hippocampus 3 days after injury
[124]	TBI	30, 60, 100 mg/kg, intraperitoneally	Ketamine exhibits neuroprotective effects by attenuating oxidative stress and apoptosis after TBI
[82]	OGD	10 mmol/L	Ketamine and propofol protected PC12 cells from OGD-induced cell apoptosis, at least partially through the SAPK/JNK signaling pathway
[203]	SNI	10 mg/kg, intraperitoneally	A single dose of ketamine could ameliorate SNI-induced depressive-like behaviors
[204]	Parkinson’s disease (PD) model	20 mg/kg, intraperitoneally	Ketamine may exert possible anti-inflammatory action in the PD model in addition to anti-dyskinetic action
[205]	Canine Model of Hypothermic Circulatory Arrest	total dose 2.85 mg/kg	Ketamine significantly reduced neurologic deficits and biomarkers of injury in canines after hypothermic circulatory arrest
[206]	Asphyxia cardiac arrest (ACA) model	40 mg/kg, intravenously	Pre-treatment with Ketamine before ACA significantly improved early survival and attenuated alterations in pH after return of spontaneous circulation (ROSC) when compared to placebo control rats

### Neurotoxicity

The neuroprotective/neurotoxic effects of ketamine seem to depend on the target, dosage, timing of application, frequency, and the presence of noxious stimulus [114–116]. Ketamine is known to have neurotoxic effects at high doses, particularly in developing neuronal models [13]. Moreover, numerous studies have shown that only dosages of ketamine above clinical concentrations could induce neurotoxicity. The above evidence suggests a dose-dependent neurotoxicity of ketamine, which may be achieved through the upregulation of p-mTOR and its downstream regulators (p-4E-BP-1 and p-p70S6K). Inhibition of the mTOR signaling pathway is protective against ketamine-induced neuronal damage in the rat hippocampus, possibly by reducing apoptosis, oxidative stress, and the concentration of calcium ions [114]. Ketamine anesthesia administered in mid-pregnancy has been shown to induce neurological damage (altered hippocampal nerve and dendritic spine density), leading to cognitive memory impairment and hippocampal neurotoxicity in the offspring via the Wnt (wingless)/β- Catenin signaling pathway [117]. Additionally, it was observed that (R, S)-ketamine and (S)-ketamine could induce the expression of HSP-70 (heat shock protein 70, a marker of neuronal damage), in rats after a single dose [118]. In an in vitro model of neural stem cell-derived neurons (NSCN), ketamine has been reported to decrease the phosphorylation of glycogen synthase kinase 3β (GSK-3β), which could lead to apoptosis [119]. These findings collectively demonstrate the neurotoxicity of ketamine in animal and cellular models.

LncRNAs and microRNAs (miRNAs) play crucial roles in the neuroprotection/neurotoxicity of ketamine. There is a substantial body of evidence suggesting that LncRNAs are involved in the neurotoxicity induced by ketamine. For instance, Yao *et al.* discovered that the overexpression of lncRNA KCNQ1OT1 significantly inhibited ketamine-induced apoptosis in PC12 cells of rats [120]. Similarly, Chen *et al.* have observed that lncRNA LINC00641 was significantly decreased in PC12 cells after ketamine treatment, and its overexpression notably mitigated apoptosis [121]. Furthermore, silencing of lncRNA IGF2AS has been found to offer protection against apoptosis in mouse neurons exposed to ketamine [122]. MicroRNAs could regulate the expression of genes at the post-transcriptional or translational level by binding to the 3ʹ untranslated region (3ʹUtr) of miRNAs. Recent studies have indicated the involvement of miRNA in the regulation of anesthesia-induced neurotoxicity. This included the down-regulation of miR-34c [123]and the up-regulation of miR-206 [120].

In a word, the neurotoxicity of ketamine is not obviously neglectable, making the usage of ketamine very careful.

### Neuroprotection

Regarding neuroprotection, the efficacy of ketamine appears to be dose-dependent. At 24 and 48 h after TBI, the high dose (60 mg/kg) of ketamine was more effective than the low dose (30 mg/kg). However, the highest dose (100 mg/kg) failed to further enhance the neuroprotective effect [124]. Various mechanisms are associated with the neuroprotection induced by ketamine, including the inhibition of inflammatory response, apoptosis, oxidative stress, excitotoxicity, and calcium overload, and the facilitation of cell proliferation, synaptic plasticity, and neuroregeneration. Exposure to ketamine (60 mg/kg) after TBI could activate the Nrf2 signaling pathway, leading to the inhibition of neuronal apoptosis and oxidative stress [124]. Additionally, ketamine has been reported to inhibit apoptosis in dentate gyrus cells and may display neuroprotective effects in hippocampal CA1 and CA2 [125]. Ketamine, as a potent NMDA receptor antagonist, could exert neuroprotective effects by alleviating excitotoxicity and calcium overload induced by excess glutamate. One study revealed that ketamine (25 mg/kg) significantly reduced status epilepticus (SE)-induced neurodegeneration and inhibited the activation of microglia by blocking NMDA 2B (GluN2B) receptors [126]. Nevertheless, a recent study has unveiled a novel NMDAR-independent mechanism of neuroprotection induced by ketamine. Ketamine could prompt the translocation of Gαs from lipid rafts to non-raft structural domains. This could lead to the elevation of intracellular cAMP levels, subsequently activating the transcriptional regulator CREB. Ultimately, this cascade may upregulate the expression of BDNF. Notably, this effect was observed even after the knockdown of the GluN1 subunit (glutamate ionotropic receptor NMDA type subunit 1) [127]. Moreover, in young mice, a single in vivo injection of ketamine, targeting GluN2B, was found to restore the density of the normal spine and synaptic plasticity [128]. In addition to the neuroprotective mechanisms mentioned earlier, ketamine plays a role in cell proliferation, synaptic plasticity, and neuroregeneration. It has been proposed that ketamine could promote hippocampal cellular proliferation following chronic brain injury and ameliorate inversion learning deficits induced by chronic constriction injury of the sciatic nerve (CCI) in mice's water maze [129]. BDNF, short for brain-derived neurotrophic factor, serves as a potent stimulator of neuronal growth and differentiation. It primarily exerted its effects by binding to the TrkB, which could regulate neuronal maturation, synapse formation, and synaptic plasticity [120]. The influence of ketamine on BDNF appears to be two-fold. On one hand, ketamine can block the BDNF levels in the hippocampus, impairing long-term memory [130, 131], On the other hand, acute administration of ketamine may enhance the expression of BDNF [132]. Finally, regarding the question of whether ketamine provides neuroprotection, a recent study has suggested that ketamine exerted neuroprotective effects by reversing the adverse effects of LPS-induced post-ischemic sepsis-like states. This potential neuroprotection was likely due to its immunomodulatory effects rather than direct neuroprotection [133].

## Etomidate

Etomidate is a non-barbiturate intravenous sedative. Limited research has explored its potential neurological repair effects. Reported studies have claimed that etomidate could inhibit cerebral metabolism, and reduce vascular-mediated inflammation, thereby exerting neuroprotective effects [134, 135]. However, contrary evidence has also suggested that etomidate may have neurotoxic effects through the nitric oxide syntheses pathway, potentially exacerbating ischemic injury [136].

## Midazolam

Midazolam, a fast-acting, short-acting benzodiazepine that variably modulates GABA-A receptors, is commonly used in anesthesia and clinical practice as a sedative, anxiolytic, hypnotic, and amnesic agent [137, 138]. Studies have investigated the neuroprotective effects of midazolam in focal cerebral ischemia and epilepsy. Some researchers pointed to its ability to inhibit seizures, neurodegeneration, and neuroinflammation when administered early (within 10 minutes) after exposure to di-isopropyl fluorophosphate (DFP) [139]. Midazolam has been found to have anti-neurotoxic effects, including reducing hydrogen sulfide-induced mortality [140], attenuating ketamine-induced toxicity in the hippocampus [141], and preventing sevoflurane-induced hippocampal neuronal toxicity [142]. Additionally, Midazolam has been reported to attenuate neuronal excitotoxicity, achieved by reuptake of excess glutamate and inhibition of glutamate release via the glutamate transporter [143, 144]. Anti-apoptotic effects also contribute significantly to midazolam's neuroprotective action. In a rat model of transient middle cerebral artery occlusion (tMCAO), midazolam was found to protect neurons in the ischemic environment by inhibiting the release of lactate dehydrogenase (LDH) and regulating the expression of apoptosis-related genes to reduce cytotoxicity and apoptosis [145]. However, while many studies support the neuroprotective effects of midazolam, some also present opposing views, suggesting that it may lack neuroprotective effects on brain tissue. For example, midazolam has been found to lack neuroprotective effects on brain tissue damage following trauma. It can impair functional recovery, and this effect cannot be mitigated by its antagonist flumazenil [146]. Midazolam has also been reported to impede neural stem cell proliferation in neonatal rats, leading to a significant decline in spatial learning memory in adult rats. Furthermore, exposure to midazolam can inhibit the proliferation of cells in the subventricular zone (SVZ) and sub granular zone (SGZ) within the hippocampus, leading to apoptosis.

## Remimazolam

Remimazolam is a novel benzodiazepine agonist targeting the GABA-A type receptor. This compound could exhibit the ability to stabilize hemodynamics, ensuring swift induction and emergence from anesthesia while exerting minimal inhibitory effects on respiration [147, 148]. Several studies have reported that remimazolam could possess the capacity to suppress cellular pyroptosis and the expression of inflammatory factors. Moreover, it has been found to effectively ameliorate neurological dysfunction following I/R injuries, lessening the extent of brain infarction and mitigating cortical neuronal damage [149]. Nevertheless, there were suggestions that remimazolam may induce neuronal apoptosis through glutamate excitotoxicity, potentially resulting in memory impairment [150].

In summary, both midazolam and remimazolam exhibit a dual nature, with both neuroprotective and neurotoxic effects. However, research on the neurological damage and repair mechanisms associated with benzodiazepines remains limited. More comprehensive animal and clinical studies, which are crucial for guiding clinical decision-making effectively, are warranted to provide robust evidence and to uncover the neuroprotective or neurotoxic mechanisms.

## The Effects of Intravenous Anesthetics on Peripheral Nerve Injuries

Compared with the widely reported neuroprotective/neurotoxic evidence of common intravenous anesthetics in CNS, the related evidence in PNS is particularly sparse. Perioperative nerve injuries (PNI) is a widely acknowledged complication associated with general anesthesia, persistently contributing to patient disability and malpractice claims [151]. One on hand, an essential consideration concerning anesthesia-induced PNI is the occurrence of such injury due to peripheral nerve blocks. On the other hand, tourniquet pain induced by intravenous regional anesthesia (IVRA) is also related to peripheral nerve injuries, of which the mechanism may be nerve ischemia and compression [152, 153]. Intravenous anesthetics often serve as an adjuvant to reduce the nerve fiber damage of peripheral nerve blocks caused by local anesthetics [14–17] and play as an effective means to treat pain [18–22]. In addition, numerous studies have indicated that the addition of Dex as an adjuvant for IVRA could also improve block characteristics, prolong intraoperative analgesia, increase the duration of both sensory and motor blockade, and accelerate the onset of sensory block. Therefore, it is essential to demonstrate the role of intravenous anesthetics on peripheral nerve damage. Next, the direct evidence on the relationship between commonly used intravenous anesthetics and PNI is presented. We will describe the role of intravenous anesthetics both in intravenous regional anesthesia and peripheral nerve block, with a particular focus on intravenous regional anesthesia, in keeping with the theme of our review.

### Dex

#### *IVRA*

Intravenous regional anesthesia** (**IVRA) is an easy and reliable anesthetic technique with a success rate, but its use is limited by tourniquet pain [154]. Tourniquet pain is described as a dull aching pain at the level of the tourniquet that increases over time despite adequate regional anesthesia. The exact etiology of tourniquet pain is unclear, but multiple mechanisms have been proposed including nerve ischemia and compression [152, 153]. These noxious stimuli activate unmyelinated C-fibers, which are responsible for dull, aching pain, as well as smaller myelinated a-delta fibers just under or near the edge of the tourniquet [153, 155].

Dex has been widely used in IVRA. Numerous studies have indicated that the addition of Dex as an adjuvant for IVRA could improve block characteristics, prolong intraoperative analgesia, increase the duration of both sensory and motor blockade, and accelerate the onset of sensory block. Finally, it is associated with improved anesthesia quality and less postoperative pain but higher sedation scores [156–158]. Dex in a dose of 1µg/kg seems to be more effective than the 0.5 µg/kg Dex in the indexes of sensory onset time, motor recovery time, sensory recovery time, and analgesic requirement [158]. However, the mechanism of action of adjunct Dex has yet to be fully established. Despite the known central α2-mediated analgesic effects of Dex, findings from an animal trial indicate that the effect of Dex is primarily due to the blockade of the hyperpolarization-activated cyclic nucleotide-gated channels in peripheral regions rather than its α1- or α2-agonistic properties in central or peripheral areas [159]. The counter-argument also suggested that this seems to be related to the intrinsic analgesic effect of systemic Dex [160], rather than its effect on nerve block duration. Furthermore, a meta-analysis demonstrated that administering Dex intravenously in regional anesthesia during surgery can potentially play a significant role in preventing postoperative delirium and postoperative cognitive dysfunction (POCD) in patients older than 60 years [161].

#### *Perineural Injection*

Dex, which could prolong the duration of sensory and motor block in animal models, is often used as an adjuvant to local anesthetics in peripheral nerve blocks [14, 15]. However, the effect of Dex on impaired peripheral nerve regeneration has been rarely studied. The preclinical data suggest that peripheral Dex may also be neuroprotective by attenuating the inflammatory response seen when the local anesthetic is applied perineurally [17, 162, 163]. A study reported that local administration of Dex around the injured peripheral nerve might facilitate peripheral nerve regeneration [164]. Similarly, Dex, when combined with bupivacaine, does not appear to increase peripheral neuronal toxicity and may reduce it [17]. Furthermore, in non-diabetic models, prior studies have demonstrated that the incorporation of Dex into perineural local anesthetics can mitigate oxidative stress and diminish the occurrence of PNI [162, 163, 165]. However, in a rat model of diabetes mellitus, another study revealed that when administered perineurally in high doses, Dex could potentiate both the duration of nerve blockade and the nerve toxicity induced by ropivacaine [166]. Dex could exert a peripheral analgesic effect (produced by inhibiting the transmission of pain signals by inhibiting Aδ and C fibers) and local analgesic effect (modulation of hyperalgesia by stimulating the α2 receptor) [23]. In CCI models [23], intramuscular injection with Dex and midazolam could attenuate the development of mechanical allodynia and thermal hyperalgesia in rats. Wu *et al*. found that the local injection of Dex to the sciatic nerve could attenuate neuropathic pain in CCI rats by reducing the expression of nerve growth factor (NGF) and sympathetic sprouting [24]. Dex may also relieve neuropathic pain by inhibiting hyperpolarization-activated cyclic nucleotide-gated currents in dorsal root ganglia (DRG) neurons [25]. Similarly, the number of swollen mitochondria in DRG neurons was significantly alleviated after Peripheral administration of Dex [26].

#### *Comparison and Summary*

At present, there is less direct evidence on the relationship between intravenous anesthetics and PNI, especially for the intravenous route of administration. In addition, intravenous administration has shown no advantages over local injection for peripheral neuroprotection. A systematic review suggested the inferiority of intravenous Dex as an adjunct to peripheral nerve block compared with perineural Dex [167]. The majority of studies reported longer durations of sensory and motor blockade, with a possible reduction in their onset times with perineural Dex. Notably, none of the trials favored intravenous administration for these outcomes. Taken together, the available evidence favors the administration of Dex via the perineural route when seeking to extend the benefits of peripheral nerve blocks [160]. Additionally, studies suggested that although intravenous administration of Dex has desirable analgesic effects, they come at the expense of the increased risk of upper respiratory obstruction, apneic episodes, bradycardia, and hypotension [168, 169]. Hence, intravenous Dex appears to be an inferior peripheral nerve block adjunct compared with perineural Dex. Instead, Kang *et al*. [170] took the opposing view that intravenous administration can not only provide the same effect as peripheral intraneural administration but also avoid the theoretical risk of nerve damage caused by perineural injection.

Here, we believe that compared to the CNS, the relationship between Dex and PNI is unclear. Although much literature has demonstrated the inferiority of intravenous Dex as an adjuvant for peripheral nerve block compared to perineural Dex, the safety of adjuvants used in the peripheral nerve has not been confirmed, and whether other components in Dex injection have neurotoxicity remains unclear. More importantly, it has not been confirmed by large-scale clinical trials, so local application should be used with caution. In the future, more efforts are needed urgently to solve this problem.

### Propofol

#### *IVRA*

An indirect piece of evidence demonstrated that the addition of propofol (20 mg) to lidocaine for IVRA could inhibit MDA levels and can be considered a useful antioxidant in this type of anesthesia [171]. However, there appears to be no evidence of direct effects of intravenous propofol in PNI.

#### *Perineural Injection*

Little data is available on the neuroprotective effects of propofol after PNI. Cao *et al*. reported that after propofol was injected into the injured sciatic nerve of mice, the expression of NF-κB in the L4–6 segments of the spinal cord on the injured side was reduced, apoptosis was decreased, nerve myelin defects were alleviated, and the nerve conduction block was lessened, finally promoting nerve repair and regeneration following sciatic nerve injury [172]. In sepsis models, propofol is currently employed to assist mechanical ventilation in patients with sepsis combined with respiratory failure, but it also may be a potential independent risk factor for severe polyneuropathy or a synergistic effect [173]. Another study proposed that anesthesia for patients with Hereditary Sensory and Autonomic Neuropathy (HSAN) II can be safely administered by using propofol alone [174].

#### *Summary*

Although the effects of propofol on the CNS have been extensively studied, little is known about its role in repair after PNI, and there is no evidence reporting the role of intravenous propofol administration in peripheral nerve injury during ITRA. Further research is needed to clarify the relationship between propofol and PNI.

### Ketamine

#### *IVRA*

Although ketamine appeared to provide some alleviation of pain [175], patients exhibited hallucinations after tourniquet deflation, when it was used together with prilocaine or lidocaine [176, 177]. For the use of ketamine in IVRA, the recommended dosages are controversial [175–177]. Gorgias *et al*. [175]recommended a dose of 0.1 µg/kg. However, compared to its systemic administration, there is no beneficial effect when using ketamine as an adjuvant for IVRA [177]. As described in this study, although the 0.3% concentration provides complete sympathetic, sensory, and motor blockade when injected into the isolated extremity, unpleasant psychotomimetic effects after the release of the tourniquet limit the usefulness of this use of ketamine. Therefore, ketamine cannot be recommended for intravenous regional anesthesia unless these unpleasant side effects are abolished or controlled by means of pharmacologic adjuvants [177].

#### *Perineural Injection*

Since the discovery of NMDA receptors on sensory afferent nerve endings in the mid-1990s, ketamine has been utilized in the peripheral compartment [178] in various forms. Topical ketamine administration can treat neuropathic pain like Complex regional pain syndrome (CRPS), postherpetic neuralgia, and intractable neuropathic pain due to ulnar nerve entrapment [18–22]. The mechanisms underlying ketamine's anti-nociceptive actions, particularly in subanesthetic doses [179] include the activation of descending inhibitory monoaminergic pain pathways and antagonism of NMDA receptors [180]. Another study indicated that sustained-release esketamine, utilizing a nanoparticle-hydrogel delivery system, can safely induce a prolonged analgesic effect in mice with spinal nerve ligation (SNL). Its mechanism may be associated with modulating the activation of astrocytes in the spinal cord and inhibiting the excitability of neurons in the DRG [181]. However, there also exists conflicting evidence that ketamine has been reported to potentiate transient receptor potential vanilloid subtype1 (TRPV1) receptor signaling in the peripheral nociceptive pathways, causing an unexpected increase in nocifensive behavior induced by capsaicin [18]. Ketamine could also serve as an adjuvant in sympathetic blocks for the management of central sensitization following PNI [182]. The action of Ketamine in the sympatholytic block is perhaps multimodal, supraspinal action by systemic absorption and peripheral action through NMDA receptors located either on the somatic nerve or in the DRG [182].

What is of paramount importance is that the toxicity of ketamine cannot be overlooked. Chronic ketamine abuse has been linked to urinary damage [175], particularly in cases of chronic and frequent high-dose exposure among ketamine abusers (dose: ≥ 0.125 g/session; frequency: 0≥ 1 day/month) [183]. Yeung *et al*. suggested that ketamine may cause degeneration of neuromuscular junctions and/or proprioceptive sensory fibers in the course of chronic addiction and the mechanisms of ketamine-induced cystitis are related to a loss of nerve fibers among the muscles of the bladder and a decline in choline acetyltransferase (a marker for cholinergic neurons) [184]. Another study reported that when administrated in ketamine, although a minor disturbance in peripheral C-fiber function did not result in increased bladder volumes, detrusor hyperreflexia has been observed in schizophrenic patients [185]. Meng *et al*. [186] conducted a study utilizing mice treated with ketamine, which demonstrated enhanced noncholinergic contractions and increased expression of P2X purinoceptor 1(P2X1). This suggests dysregulation of purinergic neurotransmission may underlie detrusor overactivity in ketamine-induced bladder dysfunction.

#### *Summary*

Overall, there is little evidence of peripheral neuroprotective or toxic effects of ketamine. Although topical administration of ketamine may prolong the analgesic effect, chronic and frequent high-dose exposure to ketamine may cause urinary damage, characterized by degeneration of neuromuscular junctions and/or proprioceptive sensory fibers. Similarly, ketamine is not recommended for ITRA because of its serious side effects. In short, in the PNS, the role of ketamine in peripheral nerve injury with ketamine is also unclear and needs to be taken seriously, as it is in the CNS.

## Interaction of Anesthetics

Previous studies have highlighted the interactions among intravenous anesthetics in the context of neuroprotection. Dex has been shown to mitigate neurotoxicity induced by various anesthetics, including ketamine, isoflurane, sevoflurane, propofol, and local anesthetics [17, 162, 163, 165, 187–189]. Dex demonstrates dose-dependent attenuation of sevoflurane-induced neurotoxicity by activating the BMP/SMAD (BMP, bone morphogenetic protein; SMAD, small mother against decapentaplegic) [39] and BDNF/TrkB [190] signaling pathways. Moreover, it mitigates the neurotoxic effects of propofol on hippocampal neurons through multiple signaling pathways, such as ERK1/2/CREB/BDNF, PI3K/Akt/Gsk3, GSK-3β/CRMP2 (CRMP2, collapsing response mediator protein 2), CDK5/CRMP2 (CDK5, cyclin-dependent kinase 5) , miR-34a/SIRT1/PI3K/Akt (SIRT1, sirtuin1), miR-377-5p/Arc (Arc, apoptosis repressor with a caspase recruitment domain), and other signaling pathways [191]. Furthermore, when combined with low doses of propofol, Dex significantly inhibited inflammatory responses, oxidative stress, and neuronal apoptosis in the hippocampal region of developing rats [192]. In addition, Dex could ameliorate hippocampal CA1 neuronal apoptosis and improve learning and memory in ketamine-injured rats [187]. Midazolam has also been found to attenuate sevoflurane-induced [142] and ketamine-induced [141] neuronal toxicity. Furthermore, the adverse effects of midazolam on neural stem cell proliferation can be antagonized by Dex [193].

## Clinical Evidence of the Neuroprotective Effects of Intravenous Anesthetics

Randomized controlled trials have provided valuable insights into the neuroprotective effects of intravenous anesthetics (Table 2). For example, the administration of Dex during craniocerebral surgery has been shown to significantly reduce the production of inflammatory mediators and markers of neuronal damage, accompanied by a reduction in oxidative stress [194]. Dex has also been shown to improve cognition and reduce the duration of post-operative delirium by increasing BDNF levels during carotid endarterectomy [9]. Additionally, Dex was considered effective in preventing the development of POCD in elderly patients [195]. Similarly, propofol has shown promising neuroprotective effects in clinical trials, improving cognitive function and reducing the incidence of POCD [196]. However, contrasting evidence suggested that the use of propofol during temporary clamping of aneurysms in subarachnoid hemorrhage (SAH) surgery may not significantly improve cognitive function [197]. In recent years, ketamine has received widespread attention for its antidepressant effects, with the majority of clinical trials focusing on its antidepressant properties as well as its effects on various psychiatric disorders [198, 199].

**Table 2:** Randomized clinical trials of intravenous anesthetics as a neuroprotective agent.

References	Patients	Surgery/diseases	Number of cases	Outcomes
[207]	Amnestic mild cognitive impairment (aMCI) (*n =* 80) and normal elderly (65–80 years old) patients (*n =* 120, control patients) who underwent a total hip joint or knee joint or shoulder joint replacement surgery	Joint replacement	80	Administration of Dex during surgery was associated with a significant reduction in postoperative delirium (POD) incidence in both normal and aMCI elderly patients
[194]	Patients older than 20 years scheduled for elective cranial surgery	Cranial surgery	160	Dex infusion combined with goal-directed hemodynamic therapy (GDHT) may mitigate neuroinflammation without undesirable hemodynamic effects during cranial surgery
[208]	Only male patients, from 40 to 60 years old, and with a Body Mass index (BMI) of 20 to 30 kg/m^2^	Lumbar discectomy	40	Dex could reverse the reduced plasma concentrations of BDNF caused by anesthetics, and this effect lasted for 24 hours after surgery
[209]	Patients with cranial glioma with ASA class II–III and BMI of 18–30 kg/m^2^	Craniotomy resection	60	Dex can significantly stabilize hemodynamics, reduce inflammation, and inhibit free radical generation
[210]	Neonates and infants (0-180 days) with D-transposition of the great arteries, ventricular septal defect, or tetralogy of Fallot	Corrective infant cardiac surgery	122	Dex results in low incidence and severity of adverse safety events in infants undergoing cardiac surgery with cardiopulmonary bypass
[211]	18 years older, diagnosed with end-stage kidney disease, undergoing kidney replacement therapy, and scheduled for donation-after-cardiac-death (DCD) kidney transplant	Cardiac -death -kidney transplant	114	24-hour perioperative Dex decreases the incidence of delayed graft function (DGF) after a DCD kidney transplant
[212]	Patients undergoing intracranial aneurysm clipping	Intracranial aneurysm clipping	60	Propofol post-conditioning (Cp 1.2 µg/mL) may protect the brain from oxidative stress injury up to 7 days post-surgery after temporary parent artery clipping, which may contribute to improvement in cognitive function
[196]	Patients aged 65–75 years scheduled for resection of an esophageal carcinoma	Esophageal carcinoma surgery	90	The incidence of POCD was higher in elderly patients undergoing major surgery under inhalational anesthesia with sevoflurane than those maintained on intravenous propofol
[213]	Adolescents (aged 13–17) with a diagnosis of major depressive disorder	Major depressive disorder	17	Ketamine is well tolerated acutely and has significant short-term (2-week) efficacy in reducing depressive symptoms compared with an active placebo
[214]	Aged 18 or older with current suicidal ideation	Suicidal ideation	156	Ketamine is rapid, safe in the short term, and has persistent benefits for acute care in suicidal patients

## Discussion

At present, the neuroprotective or neurotoxic effects of intravenous anesthetics are mainly focused on the CNS, and there are few reports on PNI or protection. In the CNS, various models of neurological injury have been studied, including cerebral ischemia-reperfusion models, traumatic brain injury models, spinal cord injury models, neonatal hypoxic-ischemic encephalopathy models, oxygen-glucose deprivation models, postoperative cognitive dysfunction models, Parkinson's and Alzheimer's disease models, and various in vitro cellular models. In these injury models, intravenous anesthetics such as Dex, propofol, and ketamine have shown neuroprotective effects, although the precise mechanisms are not fully understood. In particular, there is considerable evidence for neurotoxicity. In the PNS, very little evidence focused on the role of intravenous anesthetics in PNI. Most of the relevant evidence focused on the possibility of using intravenous anesthetics as an adjuvant to reduce the damage caused by local anesthetics during peripheral nerve blocks and the possibility of the treatment of neuropathic pain. However, very few studies have focused on ITRA. In addition, there is debate about the merits of intravenous versus perineural administration. In the future, a large number of studies are needed to investigate the relationship between intravenous anesthetics and PNI, both by intravenous administration and by perineural injection.

In conclusion, previous studies have shown that Dex has a neuroprotective effect, but the neuroprotective/neurotoxic effects of propofol, ketamine, midazolam, and remimazolam are controversial. Therefore, there is no definitive conclusion as to whether intravenous anesthetics have neurotoxic or neuroprotective effects. What's more, despite the demonstration of neuroprotective effects in numerous animal and cellular models, only a small number of clinical trials have focused on the neuroprotective effects of intravenous anesthetics. In addition, the dose and duration of intravenous anesthesia in animal studies are very different from those used in human anesthesia, making it very difficult to generalize findings from animal studies to clinical practice. Secondly, many studies remain limited and observations are often one-dimensional. Therefore, the evidence for neuroprotection or toxicity of intravenous anesthetics is not convincing. More importantly, it's well known that anesthesia is often associated with surgery, and the effect of intravenous anesthetics on neurological function may also be influenced by surgical factors. Therefore, it is difficult to exclude surgical factors to accurately discuss the effect of intravenous anesthesia on nerve function. Similarly, hypotension during surgery, hypoxia, body temperature, patient age, and physical function are also important confounding factors that cannot simply be ignored. In addition, many preclinical studies have shown that the neuroprotective/neurotoxic effects of intravenous anesthetics are dependent on dose, time, and interval of administration. What makes this topic interesting is that individual neurological vulnerability is also an important factor, suggesting that anesthesia is only a predisposing factor [200]. Therefore, whether intravenous anesthetics are neuroprotective or neurotoxic should be judged based on overall and long-term outcomes and not as an isolated phenomenon within the overall picture. That is, one should consider the "yin and yang" balance of intravenous anesthetics in daily clinical practice, which, if well implemented, will benefit patients from "precision" anesthesia.

At present, it is generally believed that the simpler anesthetics used, the shorter time, and the fewer anesthetic times could alleviate the neurotoxicity of intravenous anesthetics, and the damage to neurological function after general anesthesia due to individual neurological susceptibility needs to be further confirmed. In view of the above factors, we believe that in the future, firstly, it is urgent to promote the translation of basic research into clinical practice. Secondly, large-scale observational studies/randomized controlled trials, more sensitive outcome measures, and strict control of confounding factors are needed to draw convincing conclusions to guide the safe and efficient use of intravenous anesthesia in clinical practice. Third, we need to exploit the neuroprotective effects of intravenous anesthesia and avoid the neurotoxic effects to better guide the clinical practice of intravenous anesthesia.

## Conclusion

This review focuses on the neuroprotective and neurotoxic effects of common intravenous anesthetics in both the CNS and PNS. The selection and use of intravenous anesthetics is a complex issue. Whether intravenous anesthetics are neuroprotective or neurotoxic is not clear. Relevant studies are limited by confounding factors, a small number of clinical trials, one-dimensional observations, etc. In the future, much effort is urgently needed to investigate the neuroprotective and neurotoxic effects of intravenous anesthetics in both the CNS and PNS. Ultimately, this will better guide the clinical use of intravenous anesthetics in neuroprotective therapy.
